# *NRXN3* Is a Novel Locus for Waist Circumference: A Genome-Wide Association Study from the CHARGE Consortium

**DOI:** 10.1371/journal.pgen.1000539

**Published:** 2009-06-26

**Authors:** Nancy L. Heard-Costa, M. Carola Zillikens, Keri L. Monda, Åsa Johansson, Tamara B. Harris, Mao Fu, Talin Haritunians, Mary F. Feitosa, Thor Aspelund, Gudny Eiriksdottir, Melissa Garcia, Lenore J. Launer, Albert V. Smith, Braxton D. Mitchell, Patrick F. McArdle, Alan R. Shuldiner, Suzette J. Bielinski, Eric Boerwinkle, Fred Brancati, Ellen W. Demerath, James S. Pankow, Alice M. Arnold, Yii-Der Ida Chen, Nicole L. Glazer, Barbara McKnight, Bruce M. Psaty, Jerome I. Rotter, Najaf Amin, Harry Campbell, Ulf Gyllensten, Cristian Pattaro, Peter P. Pramstaller, Igor Rudan, Maksim Struchalin, Veronique Vitart, Xiaoyi Gao, Aldi Kraja, Michael A. Province, Qunyuan Zhang, Larry D. Atwood, Josée Dupuis, Joel N. Hirschhorn, Cashell E. Jaquish, Christopher J. O'Donnell, Ramachandran S. Vasan, Charles C. White, Yurii S. Aulchenko, Karol Estrada, Albert Hofman, Fernando Rivadeneira, André G. Uitterlinden, Jacqueline C. M. Witteman, Ben A. Oostra, Robert C. Kaplan, Vilmundur Gudnason, Jeffrey R. O'Connell, Ingrid B. Borecki, Cornelia M. van Duijn, L. Adrienne Cupples, Caroline S. Fox, Kari E. North

**Affiliations:** 1 Department of Neurology, Boston University School of Medicine, Boston, Massachusetts, United States of America; 2 Department of Internal Medicine, Erasmus Medical Center, Rotterdam, The Netherlands; 3 Department of Epidemiology, University of North Carolina at Chapel Hill, Chapel Hill, North Carolina, United States of America; 4 Department of Genetics and Pathology, Uppsala University, Uppsala, Sweden; 5 Laboratory of Epidemiology, Demography, and Biometry, Intramural Research Program, National Institute on Aging, Bethesda, Maryland, United States of America; 6 Division of Endocrinology, Diabetes, and Nutrition, University of Maryland School of Medicine, Baltimore, Maryland, United States of America; 7 Medical Genetics Institute, Cedars-Sinai Medical Center, Los Angeles, California, United States of America; 8 Department of Genetics, Washington University School of Medicine, St. Louis, Missouri, United States of America; 9 Heart Preventive Clinic and Research Institute, Icelandic Heart Association, Kopavogur, Iceland; 10 University of Iceland, Reykjavik, Iceland; 11 Division of Epidemiology, Mayo Clinic, Rochester, Minnesota, United States of America; 12 Human Genetics Center and Institute of Molecular Medicine, University of Texas Health Science Center, Houston, Texas, United States of America; 13 Department of Medicine and Epidemiology, Johns Hopkins University, Baltimore, Maryland, United States of America; 14 Division of Epidemiology and Community Health, University of Minnesota, Minneapolis, MN, United States of America; 15 Department of Biostatistics, University of Washington, Seattle, Washington, United States of America; 16 Department of Internal Medicine, University of Washington, Seattle, Washington, United States of America; 17 Department of Epidemiology, Medicine, & Health Services, University of Washington, Seattle, Washington, United States of America; 18 Department of Epidemiology and Biostatistics, Erasmus University Medical Center, Rotterdam, The Netherlands; 19 Department of Public Health Sciences, University of Edinburgh Medical School, Edinburgh, Scotland, United Kingdom; 20 Institute of Genetic Medicine, European Academy Bozen/Bolzano,Bolzano, Italy; 21 Department of Neurology, University of Lübeck, Lübeck, Germany; 22 Department of Neurology, Central Regional Hospital, Bolzano, Italy; 23 Croatian Centre for Global Health, University of Split Medical School, Split, Croatia; 24 Institute for Clinical Medical Research, University Hospital “Sestre Milosrdnice,” Zagreb, Croatia; 25 Human Genetics Unit, Institute of Genetics and Molecular Medicine, Edinburgh, Scotland, United Kingdom; 26 Department of Biostatistics, Boston University School of Public Health, Boston, Massachusetts, United States of America; 27 Program in Genomics and Divisions of Endocrinology and Genetics, Harvard Medical School, Boston, Massachusetts, United States of America; 28 Division of Prevention and Population Sciences, National Heart, Lung, and Blood Institute, Bethesda, Maryland, United States of America; 29 Division of Intramural Research, National Heart, Lung and Blood Institute, Framingham Heart Study, Framingham, Massachusetts, United States of America; 30 Boston University School of Medicine, Boston, Massachusetts, United States of America; 31 The Framingham Heart Study, Framingham, Massachusetts, United States of America; 32 Department of Clinical Genetics, Erasmus University Medical Center, Rotterdam, The Netherlands; 33 Department of Epidemiology and Population Health, Albert Einstein College of Medicine, Bronx, New York, United States of America; 34 Division of Endocrinology, Metabolism, and Diabetes, Department of Medicine, Harvard Medical School, Boston, Massachusetts, United States of America; Wellcome Trust Sanger Institute, United Kingdom

## Abstract

Central abdominal fat is a strong risk factor for diabetes and cardiovascular disease. To identify common variants influencing central abdominal fat, we conducted a two-stage genome-wide association analysis for waist circumference (WC). In total, three loci reached genome-wide significance. In stage 1, 31,373 individuals of Caucasian descent from eight cohort studies confirmed the role of *FTO* and *MC4R* and identified one novel locus associated with WC in the neurexin 3 gene [*NRXN3* (rs10146997, p = 6.4×10^−7^)]. The association with *NRXN3* was confirmed in stage 2 by combining stage 1 results with those from 38,641 participants in the GIANT consortium (p = 0.009 in GIANT only, p = 5.3×10^−8^ for combined analysis, n = 70,014). Mean WC increase per copy of the G allele was 0.0498 z-score units (0.65 cm). This SNP was also associated with body mass index (BMI) [p = 7.4×10^−6^, 0.024 z-score units (0.10 kg/m^2^) per copy of the G allele] and the risk of obesity (odds ratio 1.13, 95% CI 1.07–1.19; p = 3.2×10^−5^ per copy of the G allele). The *NRXN3* gene has been previously implicated in addiction and reward behavior, lending further evidence that common forms of obesity may be a central nervous system-mediated disorder. Our findings establish that common variants in *NRXN3* are associated with WC, BMI, and obesity.

## Author Summary

Obesity is a major health concern worldwide. In the past two years, genome-wide association studies of DNA markers known as SNPs (single nucleotide polymorphisms) have identified two novel genetic factors that may help scientists better understand why some people may be more susceptible to obesity. Similarly, this paper describes results from a large scale genome-wide association analysis for obesity susceptibility genes that includes 31,373 individuals from 8 separate studies. We uncovered a new gene influencing waist circumference, the neurexin 3 gene (*NRXN3*), which has been previously implicated in studies of addiction and reward behavior. These findings lend further evidence that our genes may influence our desire and consumption of food and, in turn, our susceptibility to obesity.

## Introduction

Body mass index (BMI) is a commonly used measure of overall adiposity. However, specific fat depots may confer differential metabolic risk. In particular, central abdominal fat, as measured by waist circumference (WC), may be more strongly associated with the development of metabolic risk factors and cardiovascular disease as compared with BMI [1]–[4]. Therefore, understanding the pathogenesis of central fat distribution may provide further insight into the relationship between adiposity, cardiometabolic risk, and cardiovascular disease.

Both genetic and environmental factors have been linked to obesity [5]. Heritability estimates for BMI and WC range from 30 to 70% in family and twin studies [6], and multiple quantitative trait loci and candidate genes have been mapped to genes for central adiposity [5]. Despite strong evidence for an underlying genetic component, genes for obesity-related traits, particularly central obesity, have been difficult to identify and replicate.

Early genome-wide association studies (GWAS) identified both *FTO* and *MC4R* as genes related to BMI and WC [7]–[10]. Many new loci have been identified in recent obesity related GWAS studies [11]–[13]. However, collectively these variants explain only a small proportion of the variation in adiposity [7]–[13]. In addition, no GWAS exist exclusively to identify genes for central fat. Thus, to identify new variants, we carried out a large-scale meta-analysis of GWAS from eight studies to detect variants associated with central body fat distribution.

## Methods

### Study Samples

Participants for the current analysis were drawn from 8 cohort studies, including the Age, Gene/Environment Susceptibility-Reykjavik Study (AGES- Reykjavik Study), the Atherosclerosis Risk in Communities Study (ARIC), the Cardiovascular Health Study (CHS), the European Special Population Network consortium (EUROSPAN), the Family Heart Study, the Framingham Heart Study, Old Order Amish (OOA), and the Rotterdam Study (RS). These groups comprise the CHARGE (Cohorts for Heart and Aging Research in Genome Epidemiology) Consortium. All participants provided informed consent. Local ethical committees at each institution approved the individual study protocols. Text S1 contains details regarding all participating cohorts.

### Imputation and Statistical Analysis

Common to all analyses were use of the raw WC measures and the assumption of an additive model; study specific details follow. Each study reported an effect allele which was meta-analyzed consistently across all studies. Results are currently presented relative to the minor G allele for the *NRXN3* SNP. In all studies except CHS, MACH (version 1.0.15 in Family Heart, Framingham, EUROSPAN and RS; version 1.0.16 in ARIC, AGES, and OOA) was used to impute all autosomal SNPs on the HapMap, using the publicly available phased haplotypes (release 22, build 36, CEU population) as a reference panel. In CHS, the program BIMBAM was used [14]. Details are provided in Table S1 regarding covariates and trait creation.

In ARIC, Framingham, and RS, sex- and either cohort-specific or study center-specific residuals were created after adjustment for age, age-squared, and smoking status. In CHS and Family Heart, linear regression models were used to adjust for age, age-squared, sex, smoking, and study center. In AGES, linear regression models using PLINK v1.04 [15] were used to adjust for age, age-squared, sex, and smoking. In the OOA the measured genotype mixed effects model was used adjusting for age, age-squared, sex and family structure based on the complete 14-generation pedigree as implemented in ITSNBN [16]. Framingham employed the linear mixed effect model for continuous traits and the generalized estimating equations for dichotomous traits in R [17] to account for family relatedness. In RS, linear regression models were run using MACH2QTL [18]. In ARIC and EUROSPAN, all regression models were run using the ProbABEL package from the ABEL set of programs [19] and in EUROSPAN genomic control [20] was used to correct standard errors of the effect estimates for relatedness among individuals. The Family Heart Study determined the effect of each SNP using linear mixed effects models to account for the siblings present in the data using SAS.

Principal components calculated using EIGENSTRAT [21] were adjusted for in the individual studies when significant in order to account for population substructure.

### Meta-Analysis

A weighted z-score approach was used to conduct meta-analyses with METAL (www.sph.umich.edu/csg/abecasis/metal/). Genomic control correction was applied to each study prior to the full meta-analysis. P-values less than 4.4×10^−7^ were considered genome-wide significant [22].

### In Silico Exchange with the GIANT Consortium

In stage 2 of our study, we conducted an *in silico* exchange of the results of 48 SNPs with the GIANT consortium. To create our list of SNPs to exchange, we first selected the top 34 SNPs from independent loci (defined as SNPs with R^2^<0.2) from our meta-analysis of WC, excluding SNPs in known loci for adiposity. An additional 14 SNPs of independent loci with a p-value<1.0×10^−5^ from a secondary list that focused on SNPs for WC with corresponding BMI p-values>0.01 were also included in an attempt to isolate genes that might be specifically associated with central fat deposition. Our *a priori* threshold for replication was a p-value<0.001 (0.05/48 SNPs) and/or reaching genome-wide significance in a combined meta-analysis. CHARGE and GIANT results were then meta-analyzed using METAL.

## Results

Table 1 presents descriptive statistics across the 8 cohorts providing data for the meta-analysis. We had a total sample size of 31,373 individuals of Caucasian descent. Participants were mostly middle-aged with ages ranging from a mean of 45 to 76 years of age.

**Table 1:** Descriptive statistics across the eight cohorts.

Cohort	N	Age (years)	% Women	Current smokers (%)	Waist Circ (cm)	BMI (kg/m^2^)
AGES	3172	76.4 (5.4)	58.0 (1840)	12.7 (402)	100.7 (12.1)*	27.1 (4.4)
ARIC	8097	54.3 (5.7)	52.8 (4276)	25.2 (2036)	96.2 (13.4)	27.0 (4.9)
CHS	3213	72.3 (5.4)	60.0 (1942)	11.0 (354)	93.6 (12.6)	26.4 (4.3)
Family Heart Study	855	55.6 (11.0)	51.5 (440)	11.9 (101)	98.6 (13.6)	27.8 (5.1)
Framingham Heart Study	7115	45.2 (10.9)	52.7 (3750)	18.8 (1338)	91.4 (15.0)	26.0 (5.1)
Old Order Amish	1134	49.6 (16.8)	48.4 (549)	9.4 (106)	88.5 (11.4)	27.0 (4.7)
Rotterdam Study	5471	69.0 (8.8)	58.6 (3205)	23.0 (1258)	90.6 (11.2)	26.3 (3.7)
EUROSPAN Consortium						
ERF (Dutch)	1239	48.3 (14.7)	60.1 (744)	43.6 (540)	87.0 (13.7)	26.7 (4.7)
CROATIAN	784	56.5 (15.3)	58.6 (459)	27.7 (217)	95.9 (11.8)	27.3 (4.3)
MICROS (South Tyrolean)	293	46.3 (15.6)	59.7 (175)	45.3 (125)	88.5 (13.3)	25.4 (5.4)

Data provided as mean (standard deviation) for continuous and % (n) for dichotomous data.

*: N = 3167 for WC by tape measure; mean (SD) of WC measured by computed tomography is 125.9(14.0) cm.

Figure S1 shows the genome-wide association results for WC in the stage 1 CHARGE-only analysis. The top SNPs for WC were in the *FTO* and *MC4R* genes (Table S3). Figure S2 shows the QQ plot for our results excluding SNPs in *FTO* and *MC4R*. For *FTO*, the top SNP was rs1558902 (p = 4.6×10^−19^). For *MC4R*, the top SNP was rs489693 (p = 3.5×10^−7^). The top results excluding SNPs in *FTO* and *MC4R* from our stage 1 meta-analysis are shown in Table 2 along with the stage 2 *in silico* replication results from the GIANT consortium; additional meta-analysis results from CHARGE are presented in Table S3. The lowest p-value on our list, for SNP rs10146997 in the *NRXN3* gene, had a stage 1 meta-analysis p-value of 6.4×10^−7^ and was confirmed in 38,641 participants from the GIANT consortium with a p-value of 0.009 and a combined p-value of 5.3×10^−8^. The *NRXN3* SNP was derived from the list of SNPs associated with WC irrespective of association with BMI. None of the other SNPs that were exchanged were confirmed in GIANT. We do note that while rs10857809 (proxy for rs10857810) in the *FAM40A* gene had a p-value of 0.003 in GIANT, the results were not direction-consistent with CHARGE and therefore did not replicate in the combined analysis.

**Table 2:** Top 48 SNPs exchanged with the GIANT Consortium, GIANT p-values, and the combined results.

Marker	Chromosome	Position	CHARGE pvalue	GIANT pvalue*	COMBINED pvalue	Nearest Gene**
rs10146997	14	79014915	6.4E-07	0.009	5.3E-08	***NRXN3***
rs981113	5	75556684	9.8E-07	0.55	3.4E-03	***SV2C***
rs7338657	13	62299289	1.1E-06	0.75	4.4E-04	*PCDH20*
rs6714750	2	136499639	1.9E-06	0.48	2.9E-03	*DARS*
rs1555967	6	51267954	1.9E-06	0.07	3.3E-06	*PKHD1*
rs4701252	5	21814911	2.5E-06	0.45	2.3E-06	***CDH12***
rs4420638	19	50114786	3.6E-06	0.80	3.8E-04	*APOC1*
rs2365642	1	199501709	4.1E-06	0.79	3.4E-03	*PKP1*
rs17008958	3	71838178	4.5E-06	0.18	5.7E-05	***EIF4E3***
rs7932813	11	7664857	4.6E-06	0.09	5.0E-06	*OVCH2*
rs569406	9	77219165	4.7E-06	0.54	3.7E-04	*OSTF1*
rs6837818	4	168112	5.2E-06	0.81	1.1E-03	*ZNF718*
rs17537900	13	42593449	7.3E-06	0.07	2.9E-03	*DNAJC15*
rs17476669	2	50579975	7.9E-06	0.27	1.1E-04	***NRXN1***
rs11857639	15	71424825	8.0E-06	0.94	3.8E-04	***HCN4***
rs3758063	8	87754664	1.2E-05	0.76	5.4E-03	***CNGB3***
rs804569	20	22099652	1.4E-05	0.29	1.7E-04	*FOXA2*
rs13002346	2	133761936	1.6E-05	0.78	1.9E-03	***NAP5***
rs7138803	12	48533735	1.6E-05	0.01	8.0E-07	*BCDIN3D*
rs17201502	12	48571829	1.7E-05	0.02	4.2E-06	***FAIM2***
rs154168	5	107078981	1.7E-05	0.86	2.0E-03	*EFNA5*
rs1324618	9	121107783	1.8E-05	0.62	0.01	***DBC1***
rs1553754	17	43918706	2.0E-05	0.05	1.2E-05	*HOXB1*
rs12971184	18	32134683	2.1E-05	0.43	0.03	***FHOD3***
rs253414	5	74992273	2.3E-05	0.47	8.0E-04	*C5orf37*
rs309193	19	52317155	2.4E-05	0.20	1.8E-04	*C19orf7*
rs12457723	18	27981438	2.4E-05	0.14	0.08	*RNF138*
rs8006194	14	88980606	2.5E-05	0.63	0.01	***FOXN3***
rs10172766	2	205587746	3.0E-05	0.30	0.01	***PARD3B***
rs11096633	2	20067535	3.1E-05	0.47	5.2E-04	***MATN3***
rs8049894	16	75371885	3.1E-05	0.67	1.9E-03	*CNTNAP4*
rs12148445	15	34703950	3.1E-05	0.60	0.01	***C15orf41***
rs9829637	3	135638752	3.5E-05	0.10	4.9E-05	*ANAPC13*
rs7666149	4	41017949	3.7E-05	0.06	2.1E-05	*LIMCH1*
rs13421140	2	1753016	4.2E-05	0.97	6.1E-03	*MYT1L*
rs4238692	16	82149934	5.8E-05	0.14	1.4E-04	***CDH13***
rs17833967	12	13846345	6.0E-05	0.46	1.2E-03	***GRIN2B***
rs1532206	3	99153367	6.2E-05	0.89	9.2E-03	***MINA***
rs6723108	2	135196450	6.2E-05	0.27	4.4E-04	*TMEM163*
rs12704232	7	85640166	7.4E-05	0.61	0.05	*GRM3*
rs12377679	9	128437576	8.0E-05	0.12	1.1E-04	***LMX1B***
rs1017643	6	156835825	9.5E-05	0.04	2.6E-05	*ARID1B*
rs6485438	11	43643194	1.3E-04	0.09	9.7E-05	*HSD17B12*
rs7116632	11	129452949	1.9E-04	0.74	0.04	***APLP2***
rs422988	1	4718977	2.4E-04	0.62	3.5E-03	***AJAP1***
rs5771623	22	47415000	2.9E-04	0.07	0.28	***FAM19A5***
rs6728666	2	216894986	5.3E-04	0.76	0.02	***MARCH4***
rs10857810***	1	110403320	1.8E-04	.003	0.97	***FAM40A***

*: GIANT sample size is 38,641.

**: Nearest reference is bolded if SNP is within the reference gene.

***: GIANT SNP is proxy rs10857809 (r^2^ = 0.92).

**Figure 1:**
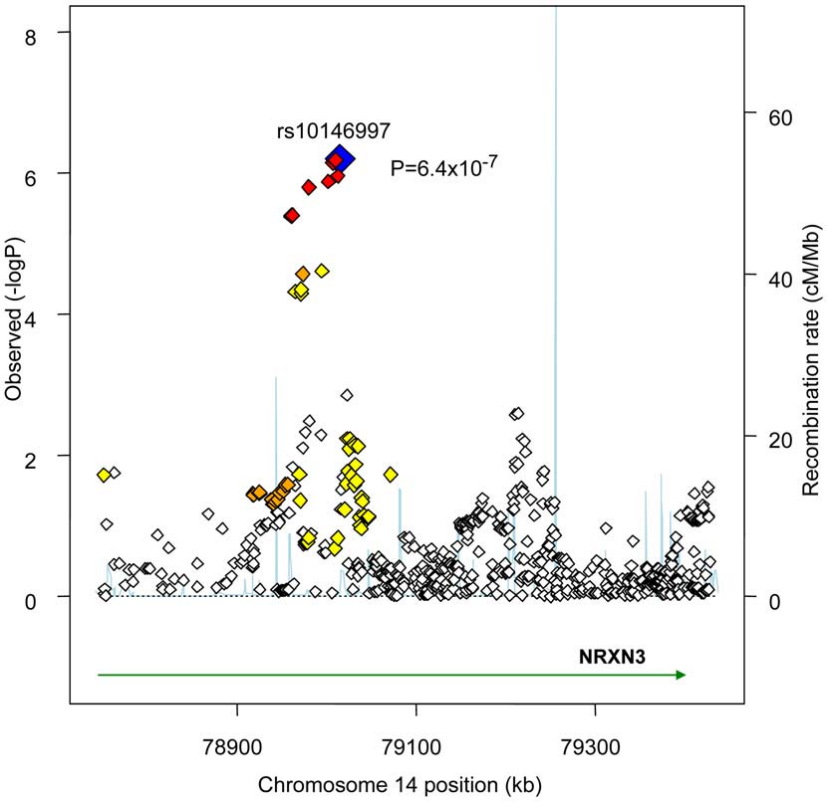
Regional Association Plot for rs10146997 on chromosome 14 in the stage 1 CHARGE-only analysis. The color scheme is red for strong linkage disequilibrium (LD; r^2^≥0.8), orange for moderate LD (r^2^≥0.5 and <0.8), yellow for weak LD (r^2^≥0.2 and <0.5) and white for limited or no LD (r^2^<0.2).

Figure 1 presents the genomic region for SNP rs10146997 (intronic) in *NRXN3*. Table 3 shows detailed results of rs10146997 in the *NRXN3* gene by contributing CHARGE study and corresponding results appear in the forest plot in Figure S3; there was no evidence for heterogeneity across the stage 1 studies (p = 0.64). The minor allele (G) frequency (MAF) for rs10146997 in our sample ranged from 0.14 in the OOA to 0.24 in the Croatians; the frequency of the *NRXN3* SNP G allele is 0.275, 1.0, 1.0, and 0.35, in Hapmap CEPH, Han Chinese, Japanese, and Yoruba populations, respectively. This SNP was genotyped in AGES, CHS, Family Heart Study, Rotterdam and all EUROSPAN studies, and imputation scores for the other studies indicated very high quality. Overall, per copy of the G allele, mean WC was increased 0.0498 z-score units (0.65 cm). Beta coefficients (in z-score units) were consistently positive in all samples except the ERF study (β = −0.0098; p = 0.86), which is most likely due to chance. Due to overlap in participants from the Framingham Heart Study and ARIC with those from the Family Heart Study, the CHARGE meta-analysis was re-run for the *NRXN3* SNP without the Family Heart Study; results were essentially unchanged (p = 6.6×10^−7^). Individual study-specific results for rs10146997 from the studies comprising the GIANT consortium can be found in Table S2.

**Table 3:** Results per copy of the G allele for rs10146997 by contributing study; beta coefficients expressed as z-scores.

Cohort	N	MAF (G)	Imputation Quality Score	Beta Coefficient	SE	p-value
AGES	3170	0.21	Genotyped	0.058	0.031	0.06
ARIC	8097	0.22	0.98	0.032	0.019	0.12
CHS	3213	0.21	Genotyped	0.103	0.030	0.00048
Family Heart Study	855	0.21	Genotyped	0.003	0.055	0.65
Framingham Heart Study	7115	0.20	1.00	0.068	0.022	0.0019
Old Order Amish	1097*	0.14	0.87	0.049	0.073	0.33
Rotterdam Study	5471	0.21	Genotyped	0.042	0.024	0.08
EUROSPAN Consortium						
ERF (Dutch)	1241	0.20	Genotyped	−0.010	0.052	0.86
Croatia	784	0.24	Genotyped	0.039	0.059	0.52
MICROS (South Tyrolean)	293	0.17	Genotyped	0.057	0.101	0.60
Meta-analysis results	31373	0.21	N/A	0.0498	0.010	6.4×10^−7^

SE = standard error; MAF = minor allele frequency.

*: Sample size reduced from 1134 because smokers excluded due to the low smoking prevalence.

Within CHARGE we also observed an association of rs10146997 with BMI (p = 7.4×10^−6^). Overall, mean BMI was increased 0.024 z-score units per G allele (0.10 kg/m^2^). When WC was additionally adjusted for BMI, the signal was completely attenuated (0.0065 z-score units per G allele; p = 0.32). The association of rs10146997 with WC was similar in women and men and in older and younger individuals (Table 4). After excluding smoking from the covariate adjustment list, results were essentially similar. Per copy of the G allele, the odds ratio of having high WC (≥88 cm in women; ≥102 cm in men) was 1.07 (95% CI 1.02–1.11; Table 4). Similarly, the odds ratio of obesity was 1.13 (95% CI 1.07–1.19).

**Table 4:** CHARGE consortium secondary analysis results per copy of the G allele for rs10146997 in 31373 individuals; beta coefficients expressed as z-scores.

	Beta Coefficient	SE	p-value
Overall	0.0498	0.010	6.4×10^−7^
Overall without adjusting for smoking	0.0460	0.010	5.6×10^−6^
Sex stratification			
Women	0.0500	0.014	4.7×10^−4^
Men	0.0427	0.013	0.001
Age stratification			
<55 years	0.0520	0.017	0.002
55+ years	0.0560	0.013	7.4×10^−6^

	Odds Ratio	95% CI	p-value
WC category*			
High WC (women ≥88 cm, men ≥102 cm)	1.07	1.02–1.11	0.003
BMI categories**			
Overweight (BMI 25 to <30)	1.03	0.98–1.07	0.250
Obese (BMI≥30)	1.13	1.07–1.19	3.2×10^−5^

*: Referent = normal WC category (women <88 cm; men <102 cm).

**: Referent = normal weight category (BMI 18.5-<25 kg/m2).

**Figure 2:**
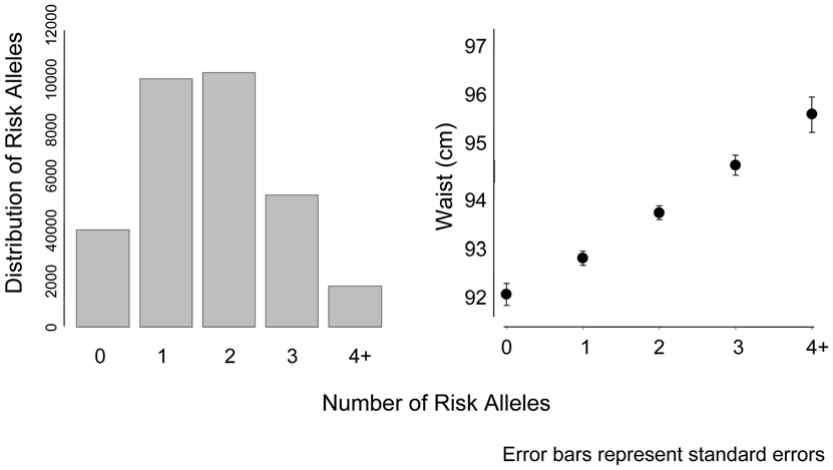
Mean waist circumference by number of risk alleles for *FTO*, *MC4R*, and *NRXN3*. Bars represent standard errors. The panel on the left represents the distribution of risk alleles in the overall sample.

We calculated a risk score of *FTO* (rs9939609), *MC4R* (rs17782313), and *NRXN3* with possible scores ranging from 0–6 risk alleles (Figure 2). Across this range, mean WC increased from 92.4 cm among those with 0 risk alleles, to 95.7 cm among those with 4 or more risk alleles. To put our findings in perspective, per copy of the effect allele, the *NRXN3* SNP resulted in a WC difference of 0.65 cm; *FTO* 0.73 cm, and *MC4R* 0.37 cm.

CHARGE consortium meta-analysis results for BMI can be found in Table S4; Manhattan and QQ plots for BMI can be found in Figure S4 and Figure S5, respectively.

## Discussion

In a discovery sample of more than 30,000 individuals from several cohort studies, we identified a novel locus in the *NRXN3* gene associated with WC. In combination with data from the GIANT consortium, the p-value for this finding exceeded our pre-defined threshold for genome-wide statistical significance. This SNP was also significantly associated with BMI and obesity. This gene has previously been associated with addiction and reward behavior, and is a compelling biologic candidate for obesity. We also confirmed the significant associations with *FTO* and *MC4R* that have previously been reported.

Although our genome-wide scan was performed for WC, the *NRXN3* SNP was also significantly associated with BMI. In secondary analyses, the signal for WC was attenuated after additionally adjusting for BMI, suggesting that this locus is most likely involved in overall adiposity and not specific to central fat deposition. Similar observations have been made for *FTO* [10] and *MC4R* [7], highlighting the inter-dependence between different measures of adiposity and the importance of performing GWAS on multiple adiposity-related traits.

The small magnitude of the effect size of the *NRXN3* variant on WC is consistent with what has previously been reported for *FTO* and *MC4R*. These findings highlight the need for large sample sizes in order to facilitate continued gene discovery for obesity-related traits. In particular, genes that emerge for waist circumference will most likely be genes for overall adiposity because of the strong correlation between the two measurements [22]. More specific measures of visceral abdominal fat depots may make it possible to isolate genes involved in regional body composition.

*NRXN3* is part of a family of central nervous adhesion molecules and is highly expressed in the central nervous system. Prior studies of *NRNX3* point towards an important role in alcohol dependence, cocaine addiction, and illegal substance abuse [23]–[26]. In addition, opioid dependence has been linked to the chromosome 14q region [23]. In mice, NRXN3 beta expression was observed in the globus pallidus when exposed to cocaine [24]. Many of the neuronal pathways in these sub-cortical regions of the brain in which NRXN3 is expressed are involved with learning and reward training [25].

Obesity and addiction may share common neurologic underpinnings [26]. Other well-replicated obesity loci, including *MC4R*, have also been shown to be associated with centrally-mediated phenomena including binge eating behavior [11],[12],[27]. Studies in mice indicate that *FTO* expression is particularly pronounced in regions of the brain known to regulate energy balance [28], and recent data suggest that variants in the *FTO* gene may regulate food intake and selection [29].

Additional research is needed to understand the association of rs10146997 with the *NRXN3* gene and to identify a causal variant. Since there are no other genes within a distance of more than several hundred kilobases of this SNP, it is unlikely that a different gene accounts for this finding. A search of publically available databases [30]–[32] did not identify an association between SNPs in *NRXN3* and gene expression.

A relationship between WC and causal variants in the *NRXN3* gene may have clinical implications. Obesity is a multifactorial trait that results from a complex interaction between genes and environment. The identification of an association between obesity and variants in a gene that has been associated with substance abuse suggests that further exploration of the role of this gene in vulnerability to addiction to food substances should be undertaken.

The strengths of this work include the large discovery sample size. The effect size was small, and achieving conventional levels of genome-wide significance required combining data from more than 70,000 participants in two large consortia. Although the confirmation with the GIANT consortium is promising, the joint p-value based on more than 70,000 participants achieved only borderline genome-wide significance. Our findings warrant the need for further replication in other ethnic groups.

We identified a SNP at a novel locus in the *NRXN3* gene associated with WC. This gene has previously been implicated in addiction and reward behavior, lending further support to the concept that obesity, in part, is a centrally-mediated disorder.

## Supporting Information

Figure S1 CHARGE consortium Manhattan plot for waist circumference.
(0.61 MB TIF)

Figure S2 CHARGE consortium QQ plot for waist circumference.
(0.44 MB TIF)

Figure S3 Forest plot for rs10146997.
(0.37 MB TIF)

Figure S4 CHARGE consortium Manhattan plot for Body Mass Index.
(0.58 MB TIF)

Figure S5 CHARGE consortium QQ plot for Body Mass Index.
(0.34 MB TIF)

Table S1 Summary of imputation and statistical analysis methods across the cohorts.
(0.08 MB DOC)

Table S2 GIANT Study-specific results for rs10146997.
(0.06 MB DOC)

Table S3 Comprehensive results from the CHARGE consortium for Waist Circumference with P<9.9×10^−6^.
(0.04 MB XLS)

Table S4 Comprehensive results from the CHARGE consortium for body mass index with P<9.9×10^−6^.
(0.04 MB XLS)

Text S1 Details of participating cohorts.
(0.07 MB DOC)
