# Supplementary figures and images for: NRXN3 Is a Novel Locus for Waist Circumference: A Genome-Wide Association Study from the CHARGE Consortium
Source: PLoS Genet. 2009 Jun 26;5(6):e1000539. doi: 10.1371/journal.pgen.1000539 (PMC2695005; doi:10.1371/journal.pgen.1000539)

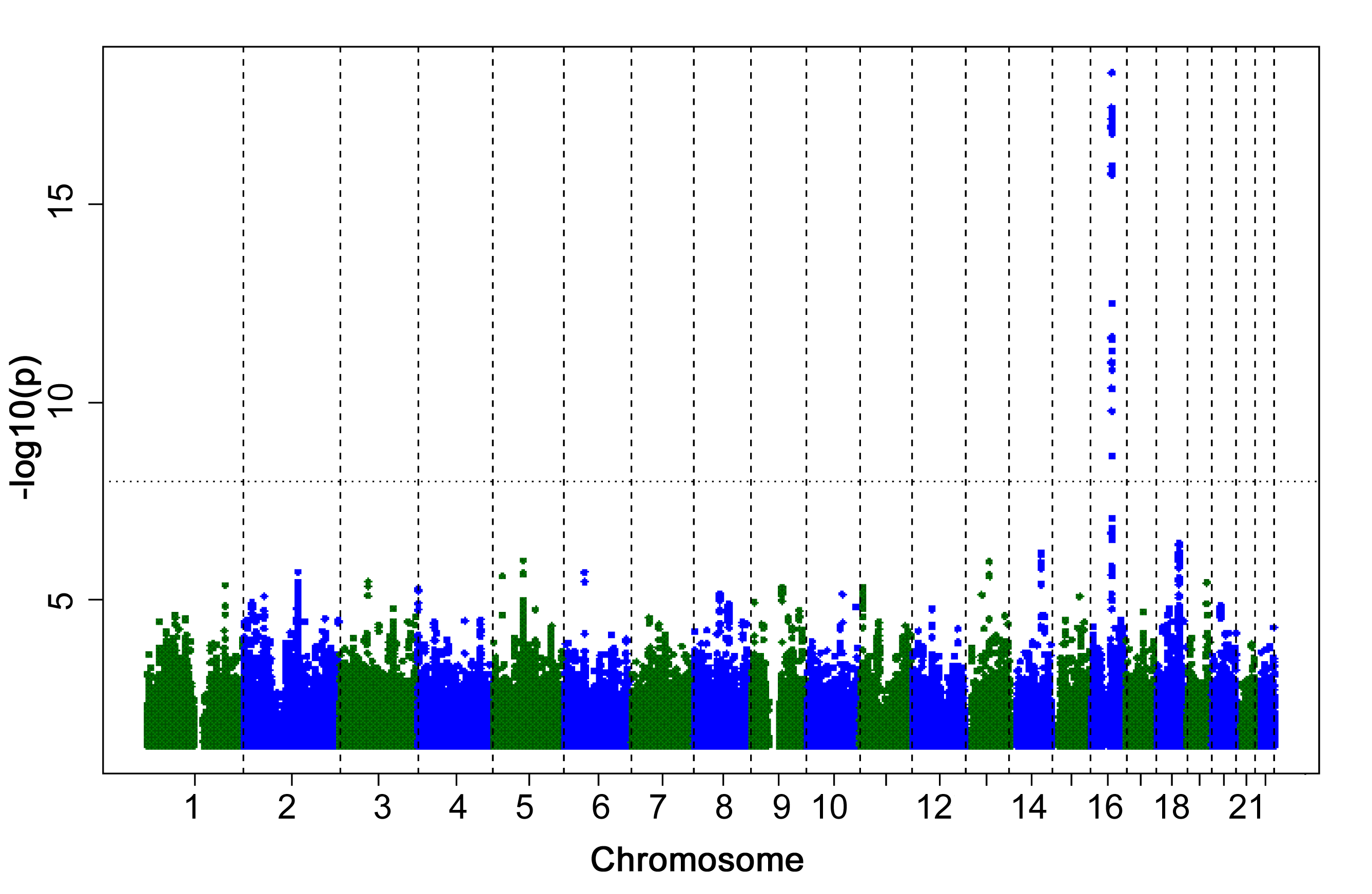

Supplement: Figure S1 — CHARGE consortium Manhattan plot for waist circumference. (0.61 MB TIF) [file pgen.1000539.s001.tif]

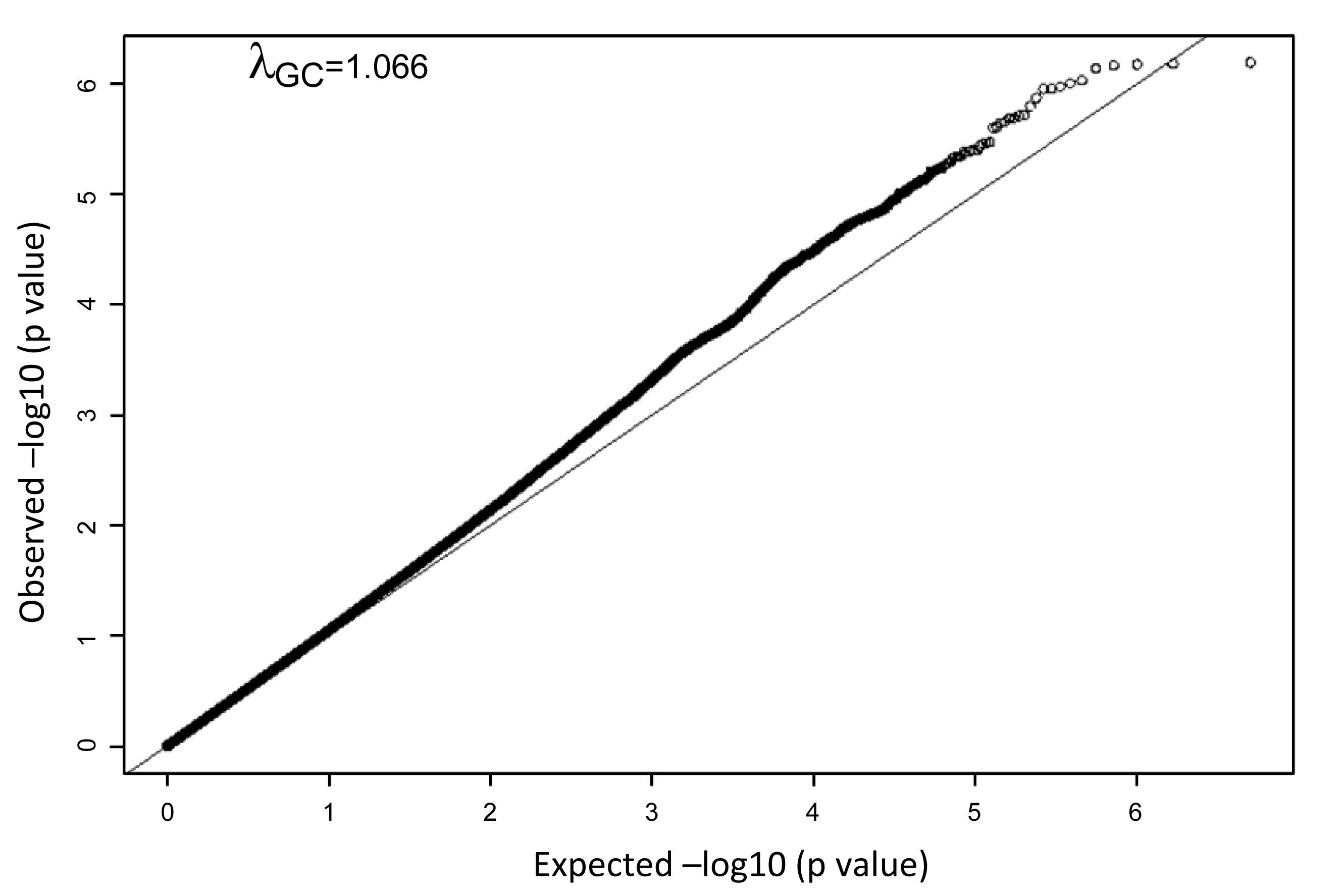

Supplement: Figure S2 — CHARGE consortium QQ plot for waist circumference. (0.44 MB TIF) [file pgen.1000539.s002.tif]

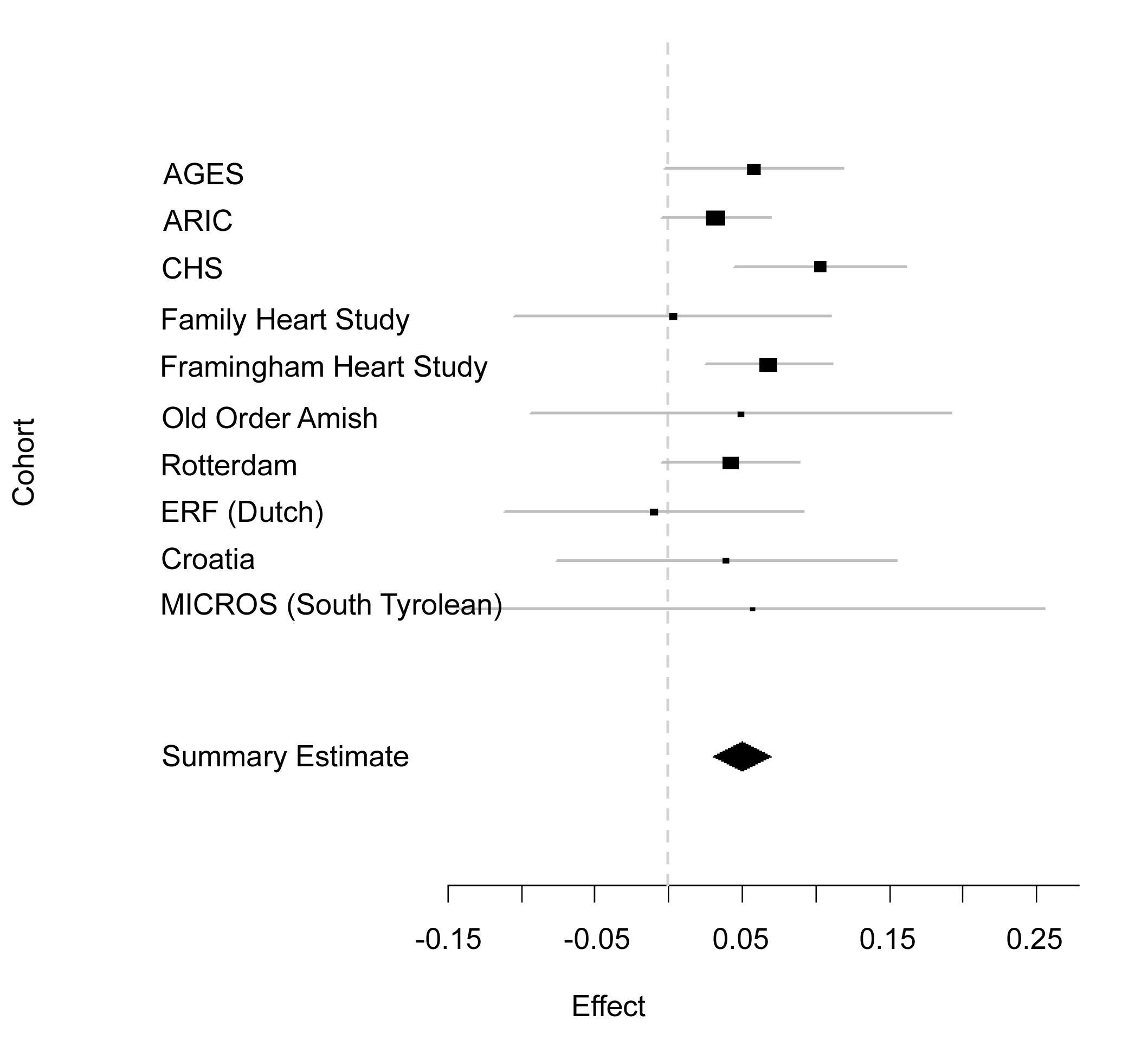

Supplement: Figure S3 — Forest plot for rs10146997. (0.37 MB TIF) [file pgen.1000539.s003.tif]

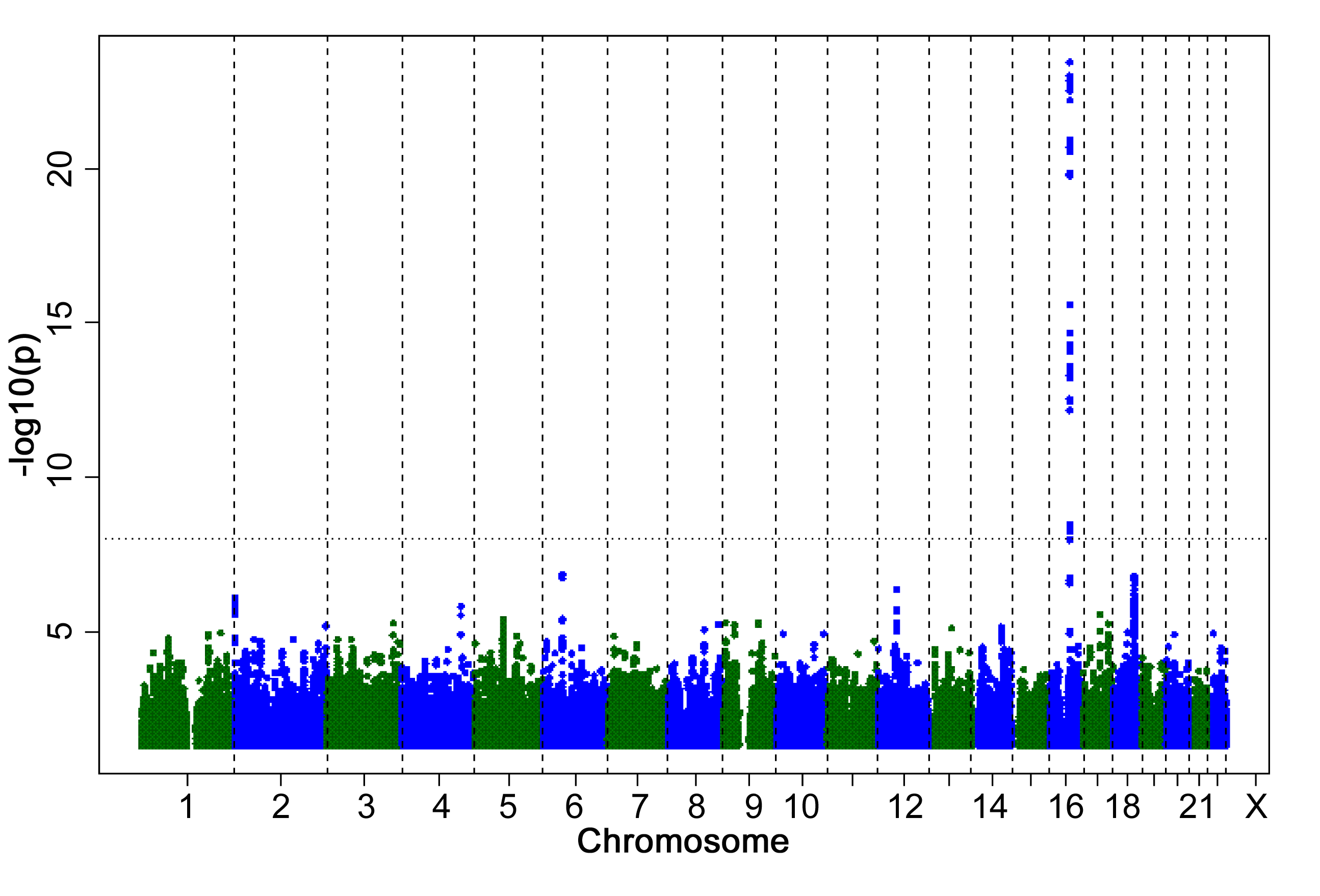

Supplement: Figure S4 — CHARGE consortium Manhattan plot for Body Mass Index. (0.58 MB TIF) [file pgen.1000539.s004.tif]

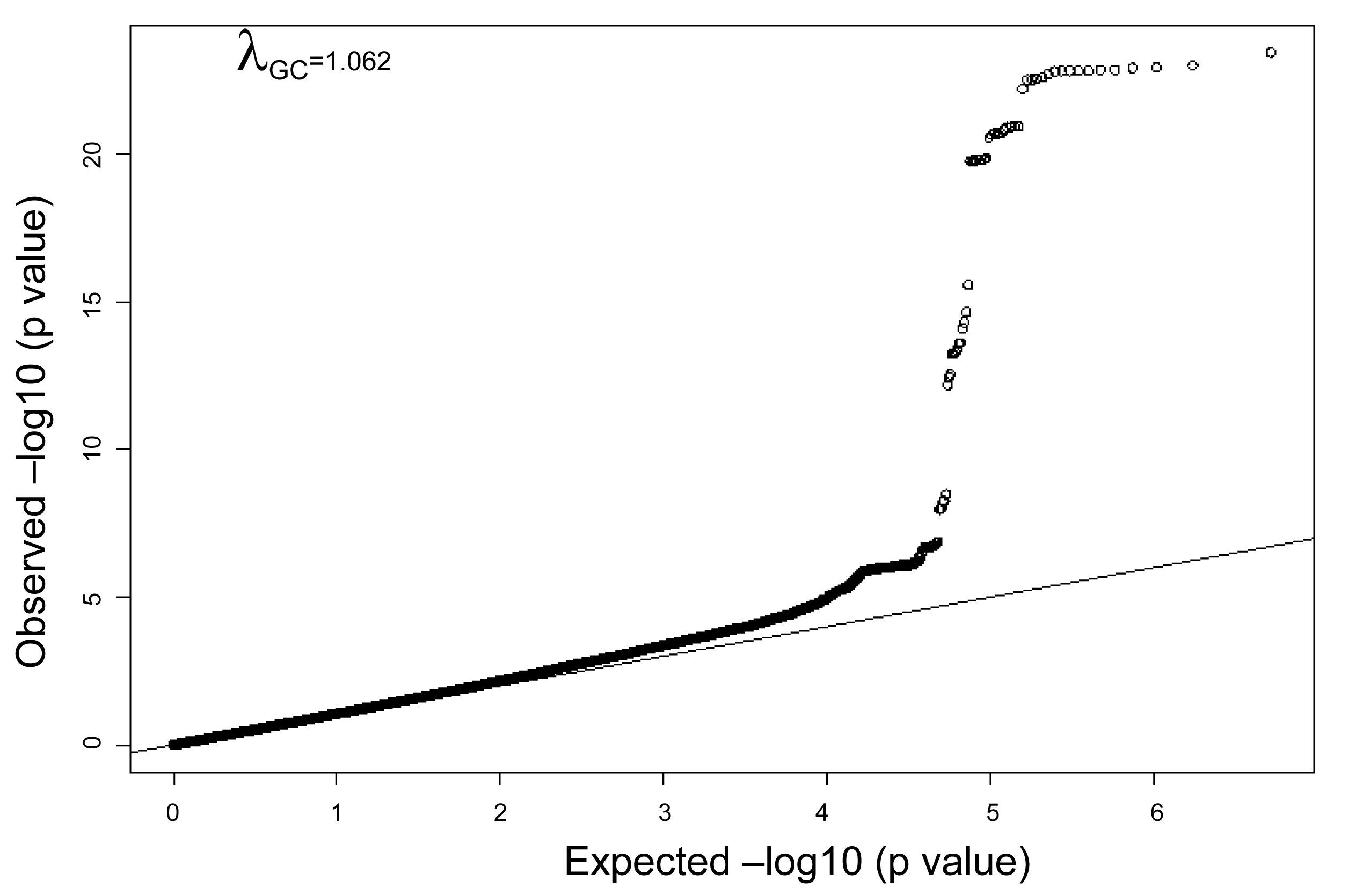

Supplement: Figure S5 — CHARGE consortium QQ plot for Body Mass Index. (0.34 MB TIF) [file pgen.1000539.s005.tif]
