# Supplementary material for: NRXN3 Is a Novel Locus for Waist Circumference: A Genome-Wide Association Study from the CHARGE Consortium
Source: PLoS Genet. 2009 Jun 26;5(6):e1000539. doi: 10.1371/journal.pgen.1000539 (PMC2695005; doi:10.1371/journal.pgen.1000539)
Supplement: Table S2 — GIANT Study-specific results for rs10146997. (0.06 MB DOC) [file pgen.1000539.s007.doc]

Table S2. GIANT Study-specific results for rs10146997

| Study | N | Beta | SE | p-value |
| --- | --- | --- | --- | --- |
| deCODE(M) | 2554 | -0.1015 | 0.034 | 0.003 |
| deCODE(W) | 3245 | -0.0775 | 0.031 | 0.019 |
| Diabetes Genetics Initiative cases(M) | 659 | 0.0550 | 0.065 | 0.403 |
| Diabetes Genetics Initiative cases(W) | 599 | -0.0440 | 0.062 | 0.484 |
| Diabetes Genetics Initiative controls(M) | 543 | -0.0330 | 0.073 | 0.656 |
| Diabetes Genetics Initiative controls(W) | 524 | 0.1640 | 0.071 | 0.023 |
| Fusion cases(M) | 621 | 0.0100 | 0.064 | 0.876 |
| Fusion cases(W) | 467 | -0.0350 | 0.071 | 0.622 |
| Fusion controls(M) | 642 | 0.0920 | 0.065 | 0.159 |
| Fusion controls(W) | 647 | 0.0250 | 0.064 | 0.698 |
| British 1958 Birth Cohort(M) | 700 | -0.0678 | 0.063 | 0.279 |
| British 1958 Birth Cohort(W) | 722 | -0.0159 | 0.068 | 0.815 |
| EPIC Obesity Study(M) | 1132 | 0.1131 | 0.053 | 0.033 |
| EPIC Obesity Study(W) | 1285 | 0.0478 | 0.047 | 0.308 |
| GSK(M) | 2560 | -0.0210 | 0.033 | 0.528 |
| GSK(W) | 2874 | 0.0027 | 0.033 | 0.935 |
| InCHIANTI(M) | 513 | -0.0200 | 0.083 | 0.810 |
| InCHIANTI(W) | 633 | 0.1630 | 0.072 | 0.024 |
| KORA(M) | 812 | -0.0290 | 0.062 | 0.641 |
| KORA(W) | 827 | -0.0250 | 0.063 | 0.692 |
| Northern Finnish Birth Cohort 1966(M) | 2254 | -0.0500 | 0.036 | 0.166 |
| Northern Finnish Birth Cohort 1966(W) | 2251 | -0.0143 | 0.035 | 0.685 |
| Nurses Health Study(W) | 1639 | -0.0080 | 0.042 | 0.849 |
| SardiNIA | 4302 | 0.0590 | 0.035 | 0.108 |
| Twins UK(W) | 1759 | 0.0020 | 0.040 | 0.961 |
| WTCCC Hypertension cases(M) | 775 | 0.0733 | 0.068 | 0.282 |
| WTCCC Hypertension cases(W) | 1177 | 0.0127 | 0.055 | 0.817 |
| WTCCC Type 2 Diabetes cases(M) | 1118 | 0.0042 | 0.050 | 0.934 |
| WTCCC Type 2 Diabetes cases(W) | 806 | -0.0036 | 0.058 | 0.952 |
| OVERALL | 38641 | 0.0247 | 0.010 | 0.009 |

M=men, W=women
