# Supplementary material for: NRXN3 Is a Novel Locus for Waist Circumference: A Genome-Wide Association Study from the CHARGE Consortium
Source: PLoS Genet. 2009 Jun 26;5(6):e1000539. doi: 10.1371/journal.pgen.1000539 (PMC2695005; doi:10.1371/journal.pgen.1000539)
Supplement: Text S1 — Details of participating cohorts. (0.07 MB DOC) [file pgen.1000539.s010.doc]

**Details of Participating Cohorts**

**Study Samples**

Participants for the current analysis were drawn from 8 cohort studies, including the Age, Gene/Environment Susceptibility-Reykjavik Study cohort (AGES-Reykjavik Study), the Atherosclerosis Risk in Communities Study (ARIC), the Cardiovascular Health Study (CHS), European Special Population Network (EUROSPAN), the Family Heart Study, the Framingham Heart Study, Old Order Amish (OOA), and the Rotterdam Study (RS). These groups comprise the CHARGE (Cohorts for Heart and Aging Research in Genome Epidemiology) Consortium. All participants provided informed consent. Local ethical committees at each institution approved the individual study protocols.

**Study Samples, Phenotype, and Genotyping**

**The AGES-Reykjavik Study**

The AGES study was originally composed of a random sample of 30,795 men and women born in 1907–1935 and living in Reykjavik in 1967 [1]. A total of 19,381 attended, resulting in 71% recruitment rate. The study sample was divided into six groups by birth year and birth date within month. One group was designated for longitudinal follow-up and was examined in all stages. One group was designated a control group and was not included in examinations until 1991. Other groups were invited to participate in specific stages of the study. Between 2002 and 2006, the AGES-Reykjavik Study re-examined 5,764 survivors of the original Reykjavik Study cohort. Waist circumference (WC) was the total abdominal circumference measured from a computerized tomography (CT) scan obtained at the mid-vertebral space between lumbar vertebrae 4 and 5 from CT scans obtained with a Siemens Somatom Sensation 4 multi-detector CT (Siemens Medical Solutions, Malvern, PA) and analyzed using specially-designed software. The correlation of this measurement with waist circumference measured with a tape at the widest body circumference is high (r=0.87, p<0.0001).

Genotyping was performed using the Illumina 370CNV BeadChip array on 3,664 participants. Sample exclusion criteria included sample failure, genotype mismatch with reference panel, and sex mismatch, resulting in clean genotype data on 3,219 individuals. Standard protocols for working with Illumina data were followed with clustering score greater than 0.4. From a total of 353,202 SNPS, 325,094 were used for imputation after exclusion of SNPs with call rate <97%, HWE deviation <1 x 10^-6^, mishap (PLINK haplotype-based test for non-random missing genotype data [2]) p<1 x 10^-9^, and mismatched positions between Illumina, dbSNP and/or HapMap.

**The ARIC study**

The ARIC study is a multi-center prospective investigation of atherosclerotic disease in a predominantly bi-racial population [3]. White and African American men and women aged 45-64 years at baseline were recruited from 4 communities: Forsyth County, North Carolina; Jackson, Mississippi; suburban areas of Minneapolis, Minnesota; and Washington County, Maryland. A total of 15,792 individuals participated in the baseline examination in 1987-1989, with follow-up examinations in approximate 3-year intervals, during 1990-1992, 1993-1995, and 1996-1998. WC was measured at the level of the umbilicus at the end of an exhalation to the nearest centimeter. Current smoking was dichotomized as yes/no according to the question “Do you now smoke cigarettes?”

ARIC Study samples were genotyped using the Affymetrix Genome-Wide Human SNP Array 6.0 (Santa Clara, California); for the current analysis only white participants were analyzed. Sample exclusion criteria included discordant with previous genotype data (n=83), genotypic and phenotypic sex mismatch (n=32), suspected first-degree relative of an included individual based on genotype data (n=297), genetic outlier as assessed by Identity by State (IBS) using PLINK [2] and >8 SD along any of the first 10 principal components in EIGENSTRAT [4] with 5 iterations (n=322). Autosomal SNPs were used for imputation after exclusion of SNPs with HWE deviation p<5 x 10‾^5^, call rate <95%, or MAF<1%.

**The Cardiovascular Health Study**

The CHS is a population-based longitudinal study of risk factors for cardiovascular disease and stroke in adults 65 years of age or older, recruited at four field centers (Forsyth County, NC; Sacramento County, CA; Washington County, MD; Pittsburgh, PA) [5]. Overall, 5201 predominantly Caucasian individuals were recruited in 1989-1990 from random samples of Medicare eligibility lists, followed by an additional 687 African-Americans recruited in 1992-1993 (total n=5,888). The CHS genome-wide association study (GWAS), which had the primary aim of studying incident cardiovascular events, focused on 3980 CHS participants who were free of clinical cardiovascular disease at study baseline, consented to genetic testing, and had DNA available for genotyping. A total of 1,908 persons were excluded from the GWAS study sample due to the presence at study baseline of coronary heart disease, congestive heart failure, peripheral vascular disease, valvular heart disease, stroke, or transient ischemic attack. CHS participants completed standardized clinical examinations and questionnaires at study enrollment and at nine annual follow-up visits. Waist circumference measurements from the baseline examination, measured at the level of the umbilicus at mid-respiration, were used for analyses. Current smoking status was self-reported at baseline. CHS Study samples were genotyped using the Illumina HumanCNV370-Duo BeadChip system. Because the other cohorts were predominantly of European descent, the African American participants were excluded from this analysis. Genotyping was successful in 3,291 Caucasian subjects. Participants were eligible for the present investigation if their genotyping was complete and they had available phenotype information. Samples with call rate <95% were excluded. A total of 306,655 autosomal SNPs were used in imputation after filtering out SNPs with HWE deviation p≤1 x 10‾^5^, call frequency ≤97%, zero heterozygote frequency, missing from dbSNP, and >1 duplicate or Mendelian inconsistency.

**The EUROSPAN populations**

Five populations are part of the EUROSPAN (European Special Population Network) project, which aims to study the genetic and environmental determinants of quantitative phenotypes of clinical importance. As inclusion criteria, the EUROSPAN populations had to a) represent local populations occupying a well defined geographic area and have limited exchange with surrounding populations enabling the inclusion of large family groups, b) have historical records enabling their genealogy and migration patterns to be defined, and c) span the geographic range and environmental diversity inhabited by European populations. For the present analysis, only the Dutch, South Tyrolean, and Croatian samples measured WC and are included in this analysis.

The South Tyrolean and Croatian populations are small populations founded by a limited number of individuals and/or have undergone a population bottleneck, followed by long isolation and no immigration [6]. The Dutch population is a recently isolated population located in the southwest of the Netherlands [7]. Despite the different demographic history of the three populations, they all have increased linkage disequilibrium (LD) compared to European populations from large urban areas [8-12].

Samples from South Tyrol were collected as part of an extended genetic study (MICROS) from settlements in Venosta Valley. The MICROS study was an extensive survey carried out in Val Venosta (South Tyrol, Italy) in 2001. Participants were from three isolated villages located in the Italian Alps, in a German-speaking region bordering with Austria and Switzerland. Due to geographical, historical and political reasons, the entire region experienced prolonged isolation from surrounding populations. Information on participants’ health status was collected through a standardized questionnaire. Laboratory data were obtained from standard blood analyses. Initially 1,175 people were enrolled in the study, which has risen to approximately 1,340 during subsequent years [13].

The Dutch samples were collected within the Erasmus Rucphen Family (ERF) study which is a family-based study that includes 3,000 inhabitants of a genetically isolated community in the south-western area of the Netherlands [8]. This study population essentially consists of one extended family of descendants from 22 related couples that lived in the isolate between 1850 and 1900.

The Croatian populations are from the villages of Vis and Komiza on the Dalmatian island of Vis. These villages have complex population histories dating back from the Illyrian period and including periods of isolation and large scale emigration [14]. Overall, 784 participants from the Croatia population were available for the current analysis.

Fasting blood samples were collected and over 200 health-related phenotypes and environmental exposures were measured in each individual. EUROSPAN DNA samples (N=4,200) were genotyped according to the manufacturer's instructions using Illumina's HumanHap300 Genotyping BeadChip. Analysis of the raw data was done in the BeadStudio software with the recommended parameters for the Infinium assay and using the genotype cluster files provided by Illumina. Sample exclusion criteria included call rate <97%, identical twins, and genetic outliers (identified by classical multidimensional scaling) resulting in a total of N=2578 (790 Dutch, 1,079 South Tyrolean, and 709 Croatian) samples for downstream analysis. SNP exclusion criteria was limited to Hardy-Weinberg Equilibrium (HWE) deviations with p <1 × 10‾^10^, resulting in 317,465, 318,049, and 318,237 in the Croatian, Dutch, and South Tyrolean populations, respectively. Autosomal SNPs were used for imputation after exclusion of SNPs with HWE deviation p≤1 x 10^-6^, MAF ≤1%, and call rate ≤98% .

**The Family Heart Study**

The Family Heart Study (https://dsgweb.wustl.edu/PROJECTS/MP1.html) was begun in 1992 with the ascertainment of 1,200 families, half randomly sampled, and half selected because of an excess of coronary heart disease (CHD) or risk factor abnormalities as compared with age- and sex-specific population rates [15]. The families, with approximately 6,000 individuals, were sampled on the basis of information on probands from four population-based parent studies: the Framingham Heart Study, the Utah Family Tree Study, and two ARIC centers (Minneapolis, and Forsyth County, NC). A broad range of phenotypes were assessed at a clinic examination in broad domains of CHD, atherosclerosis, cardiac and vascular function, inflammation and hemostasis, lipids and lipoproteins, blood pressure, diabetes and insulin resistance, pulmonary function, and anthropometry. Approximately 8 years later, study participants belonging to the largest pedigrees were invited for a second clinical exam. A total of 2,767 Caucasian subjects in 510 extended families were examined.

A two-stage design was adopted for the GWAS. In the first stage, 1016 subjects were chosen, equally distributed between the upper and lower quartiles of age- and sex-adjusted values for coronary artery calcification, assessed by CT scan. These subjects were chosen to be largely unrelated; 34% of the subjects were from unique families, while 200 other subjects had 1 or more siblings selected into the sample, yielding a sample of 465 unrelated subjects. The remaining family members (N=1,753) will be genotyped in the second stage for replication of the top hits from the first stage. The results presented here represent those for the analysis of the first-stage case-control sample for anthropometric variables assessed in exam 1.

Body mass index (BMI) was calculated as weight (in kg) divided by the square of height (in meters), while waist circumference (WC) was taken at the level of the umbilicus. All subjects were genotyped on the Illumina HumMap 550 chip. Sample exclusion criteria included technical errors (n=15), call rates <98% (n=19), and discrepancies between reported sex and sex-diagnostic markers (n=5). Of the 547,353 SNP markers available, 456,293 were used as a framework map for imputation, after excluding 730 for deviations from Hardy-Weinberg equilibrium (p<1 x 10^-6^), 22,256 with MAF <1%, and 28,822 markers not in HapMap.

**The Framingham Heart Study**

The Framingham Heart study is an ongoing three-generational prospective cohort study that began in 1948 with the recruitment of 5,209 subjects that were the adult members between the ages of 28 and 62 years from the town of Framingham Massachusetts. This original cohort has been examined every two years since that date for a total of 29 exams [16,17]. In 1971, the Offspring cohort, consisting of 5,124 offspring of the original cohort members and the offspring spouses, was recruited and have been examined every four to 8 years since, for a total of 8 exams [18,19]. Finally, in 2002, children of the offspring cohort, the Third Generation, were recruited (n=4095) [20]. The Framingham Heart Study was initiated to study cardiovascular disease and its risk factors. A broad range of phenotypes have been collected, and are publicly available at the dbGaP website (http://www.ncbi.nlm.nih.gov/projects/gap/cgi-bin/study.cgi?study_id=phs000007.v2.p1) as part of the SHARe initiative.

For this analysis, waist circumference (WC) was derived from the first available offspring (exam 4) and third generation (exam1) examinations and was measured at the level of the umbilicus to the nearest quarter-inch. Current smoking was defined as smoking at least one cigarette per day in the past year, and was dichotomized as present/absent. Framingham Heart Study samples were genotyped using the Affymetrix GeneChip Human Mapping 500K array set and the 50K supplemental array set focused on coding SNPs and SNPs tagging protein-coding genes (Santa Clara, California). Sample exclusion criteria included call rate <97% (n=767) and a per subject heterozygosity > ± 5 standard deviations from the mean (n=24). Finally, 2 participants were excluded for excessive Mendelian errors, resulting in 8,481 participants included in these analyses. From a total of 534,982 genotyped autosomal SNPs in Framingham, 378,163 SNPs were used in imputation after filtering out 15,586 SNPs (Hardy-Weinberg p<1 x 10‾^6^), 64,511 SNPs (missingness >0.03), 45,361 SNPs (mishap p<1 x 10‾^9^), 4,857 SNPs (>100 Mendel errors), 67,269 SNPs (frequency <0.01), 2 SNPs (due to strand issues upon merging data with HapMap), and a further 13,394 SNPs that were not present on HapMap.

**The Old Order Amish**

The Old Order Amish (OOA) of Lancaster Pennsylvania are relatively homogenous in terms of both genetic ancestry and lifestyle characteristics. In 1727, a small number of OOA emigrated from Switzerland to eastern Pennsylvania [21,22]. Currently, the Amish in Lancaster County have expanded remarkably to over 30,000 [23]. Since 1995, the OOA have been recruited to study the genetics of a number of complex diseases and traits. These studies include the Amish Family Diabetes Study [24,25], the Amish Family Osteoporosis Study (AFOS) [26,27], the Amish Family Longevity Study (AFLS) [28], the Amish Heredity and Phenotype Intervention Heart Study (HAPI) [29] and the Amish Family Calcification Study [30]. To date, a total of approximately 4,000 adult Amish subjects have been recruited, which represents approximately 30% of the entire adult Lancaster County population.

For the current analysis, we included 1134 participants from HAPI and AFLS who have been successfully genotyped. Study subjects were aged 20 years and older and were relatively healthy. Details of the recruitment procedures and study protocols have been published previously [28,29]. Waist circumference was measured to the nearest 0.1 cm in the horizontal plane at the narrowest part of the torso as observed anteriorly between the last rib and the iliac crest. Relatively few Amish individuals smoke (20% of men and no women), and among smokers, the intensity of smoking is modest; smoking was therefore not included as a covariate in analyses. Samples were genotyped using the Affymetrix GeneChip Human Mapping 500K array set including a total of 500,568 SNPs (Santa Clara, California). A total of 338,598 autosomal SNPs were used for imputation after applying the filters: (1) not in HapMap, (2) frequency <0.01, (3) Hardy-Weinberg p<1 x 10‾^6^, and (4) missingness >0.05.

**The Rotterdam Study**

The Rotterdam Study is an ongoing prospective, population-based cohort study among 7,983 persons aged 55 years and older, living in Ommoord, a district of Rotterdam, The Netherlands. The study was designed to investigate the incidence and determinants of chronic disabling diseases. The study is composed of an outbred ethnically homogeneous population of Dutch Caucasian origin. Rationale and design have been described previously [31,32]. At baseline (1990–1993), all participants were interviewed and subsequently underwent extensive physical examination. For this analysis, waist circumference from the baseline examination was used and was measured at the level midway between the lower rib margin and the iliac crest with participants in standing position without heavy outer garments and with emptied pockets, breathing out gently. Smoking status was assessed during the interview and subjects were classified as current smokers or non-smokers of cigarettes.

Rotterdam Study samples were genotyped using the Infinium II HumanHap550K Genotyping BeadChip version 3 (Illumina). All participants of the original Rotterdam Study cohort with proper quality DNA samples (n=6449) were genotyped with the array. This study is composed of 5471 genotyped individuals with available phenotypic information. Poorly performing samples with low call rate and 10th percentile Genecall score were excluded prior to calling genotypes. Sample exclusion criteria included call rate below 97.5% (n=209), excess autosomal heterozygosity >0.336 ~false discovery rate<0.1% (n=21), mismatch between called and phenotypic gender (n=36), and outliers identified by the IBS clustering analysis (see below) clustering >3 standard deviations away from the population mean (n=102) or IBS probabilities >97% (n=129). After exclusions, a total of 5,974 samples were analyzed. SNP exclusion criteria included MAF <0.01 (n=24,977), SNP call rate <0.98 (n=811) and Hardy-Weinberg deviations with p<1 x 10‾^6^. After exclusions, 530,683 (94.5%) of all available SNPs were available for analysis, with an average call rate of 99.5%. A total of 512,349 SNPs were used in imputation after filtering out missingness ≥0.02, MAF <0.01, and Exact HWE p-value<1 x 10‾^6^.

**Supplemental Figure Legends**

Figure S1. CHARGE consortium Manhattan plot for Waist Circumference

Figure S2. CHARGE consortium QQ plot for Waist circumference

Figure S3. Forest plot for rs10146997

Figure S4. CHARGE consortium Manhattan plot for Body Mass Index

Figure S5. CHARGE consortium QQ plot for Body Mass Index

**References**

1. Harris TB, Launer LJ, Eiriksdottir G, Kjartansson O, Jonsson PV, et al. (2007) Age, Gene/Environment Susceptibility-Reykjavik Study: multidisciplinary applied phenomics. Am J Epidemiol 165: 1076-1087.

2. Purcell S, Neale B, Todd-Brown K, Thomas L, Ferreira MA, et al. (2007) PLINK: a tool set for whole-genome association and population-based linkage analyses. Am J Hum Genet 81: 559-575.

3. (1989) The Atherosclerosis Risk in Communities (ARIC) Study: design and objectives. The ARIC investigators. Am J Epidemiol 129: 687-702.

4. Price AL, Patterson NJ, Plenge RM, Weinblatt ME, Shadick NA, et al. (2006) Principal components analysis corrects for stratification in genome-wide association studies. Nat Genet 38: 904-909.

5. Fried LP, Borhani NO, Enright P, Furberg CD, Gardin JM, et al. (1991) The Cardiovascular Health Study: design and rationale. Ann Epidemiol 1: 263-276.

6. Marroni F, Pichler I, De Grandi A, Beu Volpato C, Vogl FD, et al. (2006) Population isolates in South Tyrol and their value for genetic dissection of complex diseases. Ann Hum Genet 70: 812-821.

7. Pardo LM, MacKay I, Oostra B, van Duijn CM, Aulchenko YS (2005) The effect of genetic drift in a young genetically isolated population. Ann Hum Genet 69: 288-295.

8. Aulchenko YS, Heutink P, Mackay I, Bertoli-Avella AM, Pullen J, et al. (2004) Linkage disequilibrium in young genetically isolated Dutch population. Eur J Hum Genet 12: 527-534.

9. Johansson A, Vavruch-Nilsson V, Edin-Liljegren A, Sjolander P, Gyllensten U (2005) Linkage disequilibrium between microsatellite markers in the Swedish Sami relative to a worldwide selection of populations. Hum Genet 116: 105-113.

10. Johansson A, Vavruch-Nilsson V, Cox DR, Frazer KA, Gyllensten U (2007) Evaluation of the SNP tagging approach in an independent population sample--array-based SNP discovery in Sami. Hum Genet 122: 141-150.

11. Vitart V, Carothers AD, Hayward C, Teague P, Hastie ND, et al. (2005) Increased level of linkage disequilibrium in rural compared with urban communities: a factor to consider in association-study design. Am J Hum Genet 76: 763-772.

12. Vitart V, Biloglav Z, Hayward C, Janicijevic B, Smolej-Narancic N, et al. (2006) 3000 years of solitude: extreme differentiation in the island isolates of Dalmatia, Croatia. Eur J Hum Genet 14: 478-487.

13. Pattaro C, Marroni F, Riegler A, Mascalzoni D, Pichler I, et al. (2007) The genetic study of three population microisolates in South Tyrol (MICROS): study design and epidemiological perspectives. BMC Med Genet 8: 29.

14. Rudan I, Campbell H, Rudan P (1999) Genetic epidemiological studies of eastern Adriatic Island isolates, Croatia: objective and strategies. Coll Antropol 23: 531-546.

15. Higgins M, Province M, Heiss G, Eckfeldt J, Ellison RC, et al. (1996) NHLBI Family Heart Study: objectives and design. Am J Epidemiol 143: 1219-1228.

16. Dawber TR, Meadors GF, Moore FE, Jr. (1951) Epidemiological approaches to heart disease: the Framingham Study. Am J Public Health Nations Health 41: 279-281.

17. Dawber TR, Kannel WB, Lyell LP (1963) An approach to longitudinal studies in a community: the Framingham Study. Ann N Y Acad Sci 107: 539-556.

18. Feinleib M, Kannel WB, Garrison RJ, McNamara PM, Castelli WP (1975) The Framingham Offspring Study. Design and preliminary data. Prev Med 4: 518-525.

19. Kannel WB, Feinleib M, McNamara PM, Garrison RJ, Castelli WP (1979) An investigation of coronary heart disease in families. The Framingham offspring study. Am J Epidemiol 110: 281-290.

20. Splansky GL, Corey D, Yang Q, Atwood LD, Cupples LA, et al. (2007) The Third Generation Cohort of the National Heart, Lung, and Blood Institute's Framingham Heart Study: design, recruitment, and initial examination. Am J Epidemiol 165: 1328-1335.

21. Cross HE (1976) Population studies and the Old Order Amish. Nature 262: 17-20.

22. McKusick VA (1978) Medical genetic study of Amish. Baltimore, MD: Johns Hopkins University Press.

23. Beiler K (1996) Church Directory of the Lancaster County Amish. Gordonsville, PA: Peqaea Publishers.

24. Hsueh WC, Mitchell BD, Aburomia R, Pollin T, Sakul H, et al. (2000) Diabetes in the Old Order Amish: characterization and heritability analysis of the Amish Family Diabetes Study. Diabetes Care 23: 595-601.

25. Fu M, Damcott CM, Sabra M, Pollin TI, Ott SH, et al. (2004) Polymorphism in the calsequestrin 1 (CASQ1) gene on chromosome 1q21 is associated with type 2 diabetes in the old order Amish. Diabetes 53: 3292-3299.

26. Streeten EA, McBride DJ, Lodge AL, Pollin TI, Stinchcomb DG, et al. (2004) Reduced incidence of hip fracture in the Old Order Amish. J Bone Miner Res 19: 308-313.

27. Streeten EA, McBride DJ, Pollin TI, Ryan K, Shapiro J, et al. (2006) Quantitative trait loci for BMD identified by autosome-wide linkage scan to chromosomes 7q and 21q in men from the Amish Family Osteoporosis Study. J Bone Miner Res 21: 1433-1442.

28. Sorkin J, Post W, Pollin TI, O'Connell JR, Mitchell BD, et al. (2005) Exploring the genetics of longevity in the Old Order Amish. Mech Ageing Dev 126: 347-350.

29. Mitchell BD, McArdle PF, Shen H, Rampersaud E, Pollin TI, et al. (2008) The genetic response to short-term interventions affecting cardiovascular function: rationale and design of the Heredity and Phenotype Intervention (HAPI) Heart Study. Am Heart J 155: 823-828.

30. Post W, Bielak LF, Ryan KA, Cheng YC, Shen H, et al. (2007) Determinants of coronary artery and aortic calcification in the Old Order Amish. Circulation 115: 717-724.

31. Hofman A, Breteler MM, van Duijn CM, Krestin GP, Pols HA, et al. (2007) The Rotterdam Study: objectives and design update. Eur J Epidemiol 22: 819-829.

32. Hofman A, Grobbee DE, de Jong PT, van den Ouweland FA (1991) Determinants of disease and disability in the elderly: the Rotterdam Elderly Study. Eur J Epidemiol 7: 403-422.
